# The global spread of Oriental Horses in the past 1,500 years through the lens of the Y chromosome

**DOI:** 10.1073/pnas.2414408121

**Published:** 2024-11-18

**Authors:** Lara Radovic, Viktoria Remer, Doris Rigler, Elif Bozlak, Lucy Allen, Gottfried Brem, Monika Reissman, Gudrun A. Brockmann, Katarzyna Ropka-Molik, Monika Stefaniuk-Szmukier, Liliya Kalinkova, Valery V. Kalashnikov, Alexander M. Zaitev, Terje Raudsepp, Caitlin Castaneda, Ines von Butler-Wemken, Laura Patterson Rosa, Samantha A. Brooks, Miguel Novoa‐Bravo, Nikos Kostaras, Abdugani Abdurasulov, Douglas F. Antczak, Donald C. Miller, Maria Susana Lopes, Artur da Câmara Machado, Gabriella Lindgren, Rytis Juras, Gus Cothran, Barbara Wallner

**Affiliations:** ^a^Department for Biological Sciences and Pathobiology, Animal Breeding and Genetics, University of Veterinary Medicine Vienna, Vienna 1210, Austria; ^b^Vienna Graduate School of Population Genetics, University of Veterinary Medicine Vienna, Vienna 1210, Austria; ^c^Albrecht Daniel Thaer-Institut, Humboldt-Universität zu Berlin, Berlin 10099, Germany; ^d^Department of Animal Molecular Biology, National Research Institute of Animal Production, Balice 32-083, Poland; ^e^All-Russian Research Institute for Horse Breeding, Ryazan 391105, Russia; ^f^Department of Veterinary Integrative Biosciences, Texas A&M University, College Station, TX 77843; ^g^Verein der Freunde und Züchter des Berberpferdes, Schmalenberg 67718, Germany; ^h^Department of Veterinary Clinical Sciences, College of Veterinary Medicine, Long Island University, Brookville, NY 11548; ^i^Department of Animal Science, University of Florida Genetics Institute, University of Florida, Gainesville, FL 32610; ^j^Genética Animal de Colombia, Bogotá 111071, Colombia; ^k^Amaltheia, Papagou 15669, Greece; ^l^Department of Veterinary Medicine and Biotechnology, Faculty of Natural Science, Tourism and Agricultural Technology, Osh State University, Osh 723500, Kyrgyzstan; ^m^Department of Biomedical Sciences, Baker Institute for Animal Health, Cornell University, Ithaca, NY 14853; ^n^Biotechnology Centre of Azores, University of Azores, Angra do Heroísmo 9700-042, Portugal; ^o^Department of Animal Biosciences, Swedish University of Agricultural Sciences, Uppsala 75007, Sweden

**Keywords:** Y chromosome, horse, phylogeography, migrations, breeding

## Abstract

Since their domestication, horses have accompanied mankind, and humans have constantly shaped horses according to their needs through stallion-centered breeding. Consequently, the male-specific portion of the Y chromosome (MSY) is extremely uniform in modern horse breeds. The majority of stallions worldwide carry MSY haplotypes (HT) attributed to an only ~1,500-y-old, so-called, “Crown” haplogroup. The predominance of the Crown in modern horse breeds is thought to represent a footprint of the vast impact of stallions of “Oriental origin” in the past millennium. Here, we report the results of a fine-scaled MSY haplotyping of large datasets of patrilines comprising 1,517 males of 189 modern horse breeds, covering a broad phenotypic and geographic spectrum. We can disentangle the multilayered influence of Oriental stallions over the last few hundred years, exposing the intense linebreeding and the wide-ranging impact of Arabian, English Thoroughbred, and Coldblood sires. Iberian and New World horse breeds contain a wide range of diversified Crown lineages. Their broad HT spectrum illustrates the spread of horses of Oriental origin via the Iberian Peninsula after the Middle Ages, which is commonly referred to as the “Spanish influence.” Our survey also revealed a second major historical dissemination of horses from Western Asia, attributed to the expansion of the Ottoman Empire. Our analysis shows that MSY analysis can uncover the complex history of horse breeds and can be used to establish the paternal ancestry of modern horse breeds.

## Significance

The horse has been one of the most important domestic animals in human culture, and the history of the horse is inextricably linked to geopolitical developments. Past migratory events, varied breeding goals, and intensive stallion-centered breeding have created a complex mosaic of ancestry. The paternally inherited Y chromosome reflects the male side of population history and offers a view on the origin and influence of stallions. We have analyzed a large collection of modern breeds and reconstructed their ancestry over the last 1,500 y of history. We identify three major recent breeding influences and highlight two fundamental historical routes by which Oriental Horses spread. Finally, we present an approach to investigate the paternal ancestry of any horse breed of interest.

The domestication of the horse initiated an unbreakable bond between horses and humans (1). The horse has been inextricably linked to the technological and cultural development of mankind (2), and the 60 million domestic and feral horses that exist today (FAO, 2022, available at https://www.fao.org/dad-is/en/, last accessed 14th June 2024) are the legacy of human history over the last 4,000 y (3).

The origin of the modern population lies in domesticated Bronze Age horses from the western Eurasian steppe, which rapidly spread throughout Eurasia from around 2,200 BCE onward (1, 4). However, recent studies based on genomic data from modern and historical horses showed the enormous genetic influence of “Oriental Horses” in the past millennium (5–7). The term Oriental Horse stands here for an ancient horse type that developed in the Middle East and spread worldwide after the Islamic conquest of Europe and Asia (from the 7th century CE) (3, 8). The oriental influence was complex and Oriental Horses reached Europe and the rest of the world over a prolonged period, from several directions and for many purposes (3, 8–10). They spread with soldiers, traders, and colonizers, and in recent centuries, Oriental Horses have been used in systematic breeding to adjust the phenotype of horses to meet particular needs (9, 10). The influence of oriental bloodlines systematically imported to Europe in the past 200 to 300 y is well documented, as is the case for horses from the Arabian Peninsula (Arabians) and the Asian steppes (Turkomans) in the 18th century (3). The earlier periods of dissemination of Oriental Horses that affected all modern horse breeds (3, 11, 12) are less understood. We can assume that the Iberian Peninsula represented one of the first gateways for Oriental Horses into Europe. From the 8th to the 15th century CE, at the time of the Islamic conquest, this region was occupied by north African Moors. The period saw a continuous exchange of horses between the Iberian Peninsula and North Africa (3, 12), leading to the development of a remarkable type of horse. The European nobility soon recognized the quality of these “Spanish horses,” and Spanish horses were actively introduced into Central Europe from the beginning of the Reconquista (the Christian campaign to reconquer the Iberian Peninsula, starting in 718 CE) (9). With the second voyage of Columbus (1493) and further colonization, Spanish horses also reached the American continent (11). Known as “Colonial Spanish horses,” the animals were first introduced to the Caribbean Islands and soon expanded following Spanish settlement. While European horse breeding in the 15th to 17th century was marked by the popularity of Spanish bloodlines (9, 11), Ottoman war horses, also considered as “Oriental,” influenced European, Asian, and African territories (8, 9, 13).

Molecular genetic methods have been widely used to elucidate the origin of modern horse breeds. However, there has been little work on the genetic legacy of horses disseminated to the Western Hemisphere, and most of it has been limited to a specific breed and a proposed Iberian origin (14–21). In addition, it was largely based on the maternally inherited mitochondrial DNA (mtDNA) or autosomal genetic variation. This seems paradoxical as from the very start horse breeding has been founded on a strong selection of stallions (5, 22–24), which has reached its peak in the past 300 y. The enormous influence of stallions makes the strictly paternally inherited, male-specific part of the Y chromosome (MSY) an ideal marker to investigate the establishment and demography of horse breeds (25, 26).

The strong selection of stallions has resulted in an extremely reduced genetic variation of the horse MSY (27–29). The majority of horse breeds from central and southern Europe, central and western Asia, and north and south America carry MSY haplotypes (HT) that cluster into a roughly 1,500-y-old haplogroup (HG) (7, 30). Based on its distribution and its time of emergence, this HG (termed as “Crown”) is believed to be the footprint of Oriental stallions that transformed the genetic landscape of the horse in the past millennium (6, 7, 30, 31). “Non-Crown” HTs have only been found in Przewalski’s horses, local Asian populations, some northern European horses, and a few island populations (7, 31). Within the Crown, the signatures of recent influential breeding sires, such as those of the three founders of English Thoroughbreds (30) and some Arabian stallions (32), have been described and their influence on north African horses (33), Mongolian horses (34, 35), Kaimanawa horses (36), Estonian native horses (37) inferred.

We have used fine-scale MSY haplotyping of modern horse breeds to create a comprehensive phylogeographic view of the distribution of MSY Crown HTs in modern horse breeds. Our results provide a key to the demographic processes that gave rise to most of today’s horse populations. We show that the spread of Crown HTs is closely related to historical developments in Europe and to intensive breeding. The current distribution of Crown lineages reveals the wave-like influence of Oriental stallions over the past millennium and shows the temporal and spatial dynamics that led to the formation of modern horse breeds.

## Results

### Crown HGs Dominate in Modern Horse Breeds.

We determined the distribution of the Crown HG (daC) in a collection of 1,517 male horses representing 189 breeds from thirteen worldwide regions by genotyping (*Materials and Methods* and *SI Appendix*, Figs. S1–S4). The dataset captured the majority of modern breeds and included Arabians, Thoroughbreds, European Coldbloods and Ponies, European and US riding horses, Iberian, North African, and Colonial Spanish horses, and numerous local riding and light draft breeds (Dataset S1 gives full details). We selected a maximum of 15 horses per breed and documented the paternal tail line (the lineage traced through the male ancestors) to represent the sire lines active in a breed.

**Fig. 1.**
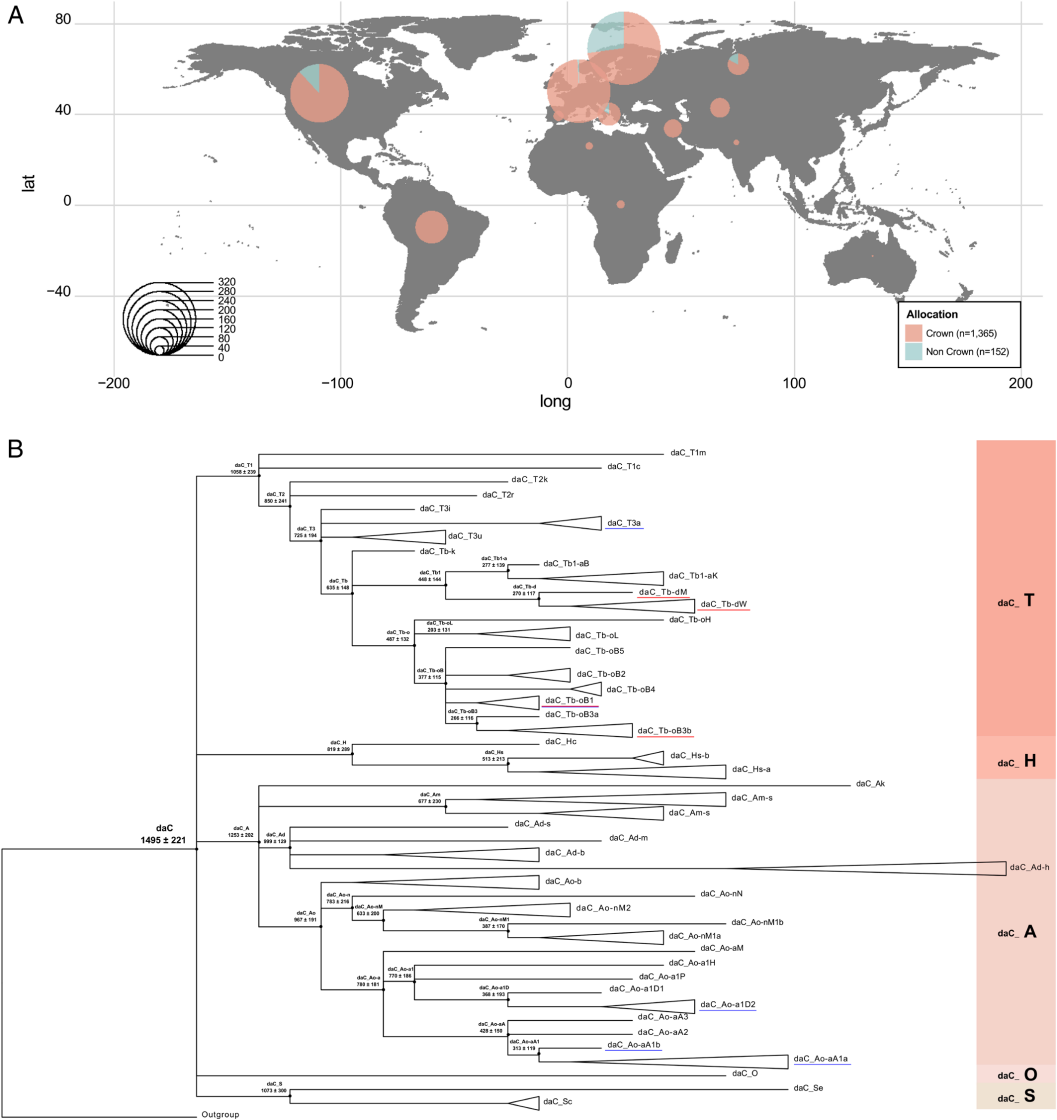
Distribution and topology of the MSY Crown HG (*A*) Distribution of the Crown HG identified from the dataset (n = 1,517; *SI Appendix*, Fig. S1). Samples from thirteen geographic regions (*SI Appendix*, Fig. S2) illustrated as pies and radiuses scaled to the number of individuals screened. (*B*) A maximum parsimony tree showing the Crown portion of the MSY phylogeny based on 512 variants in 120 males from ref. 7; rooted with a non-Crown Icelandic horse. The five clearly separated major daC HGs are represented in pink on the right [daC_T (n = 53), daC_H (n = 7), daC_A (n = 56), daC_O (n = 1), and daC_S (n = 3)]. Estimates of divergence time and CI are given left of the branching points; precise estimates of divergence are given in *SI Appendix*, Table S1. Previously determined Thoroughbred-specific HTs are underlined in red and HTs characteristic of Arabians in blue (30, 32). Dataset S3 gives full details of samples and HTs.

The results confirmed the predominance of the Crown HG, which clustered 90% of the samples. It was detected in 177 of the 189 horse breeds and only 21 breeds (152 samples) showed non-Crown HTs. The Crown HG was distributed worldwide (Fig. 1*A*), while we only found non-Crown HTs in northern regions (north America, north Europe, and north Asia), and in some island populations (central and southeast Europe), in concordance with previous findings (7, 21, 30, 31, 34).

We then reconstructed a published horse MSY phylogeny, based on 2,678 single-nucleotide polymorphisms (SNPs) from ref. 7, and dated the most important branching points in the Crown under the assumption of a molecular clock. The most recent common ancestor (MRCA) of the Crown was dated to 1,495 ± 221 y BP (*Materials and Methods* and *SI Appendix*, Table S1), a value that corresponds to independent dating estimates based on the molecular clock (30) and ancient DNA (7). We distinguished five major HGs within the Crown with their approximate MRCA 800 to 1,200 y BP. Fig. 1*B* presents a parsimony tree, representing all Crown HTs distinguished to date (details on samples in Dataset S3) and with dating estimates on branching points.

The next step was to construct a downscaled structure of a published horse Y phylogeny (7), based on 124 selected, mainly Crown, HT-determining variants (DV) (*SI Appendix*, Figs. S3 and S4 and Dataset S2). We used this structure as a backbone for a detailed determination of HTs (*Materials and Methods*, *SI Appendix*, Figs. S3 and S4, and Dataset S2) by genotyping the set of 1,517 male horses. Of the 1,365 Crown samples, 965 (71%) carried a predefined HT (57 Crown HTs were detected), while 400 (29%) were allocated to inner nodes of the backbone topology. We observed such inner node groupings at 33 branching points, so a significant fraction of the samples in our dataset carries Crown HTs that are not yet resolved due to the lack of private variants. We labeled the internal HTs with the branching point followed by an asterisk (explained in *SI Appendix*, Fig. S3; e.g., daC*, daC_Ao*, daC_T1*). The HT for each sample is given in *SI Appendix*, Fig. S5 and Dataset S1 presents an overview of HGs detected across breeds and regions.

### MSY Traces of Recent Breeding in Arabian- and Thoroughbred-Influenced and in Coldblood Breeds.

We divided the 1,365 Crown samples from 177 breeds into seven major groups based on type and breed history, concordance with breed allocation reported with autosomal markers (38, 39), and paternal tail line from the pedigree (*Materials and Methods*). The breed groups were Arabians including Arabian lines in other breeds (n = 69), Thoroughbreds including Thoroughbred lines in other breeds (n = 93), Coldbloods (n = 195 in Crown), breeds with recorded Spanish influence (n = 345), European and US riding breeds (n = 146), local riding and light draft breeds from Europe, the United States, and other colonial territories (n = 323 in Crown), and local riding and light draft breeds from Asia (n = 194 in Crown) (see *SI Appendix*, Fig. S1 and Dataset S1 for details).

**Fig. 2.**
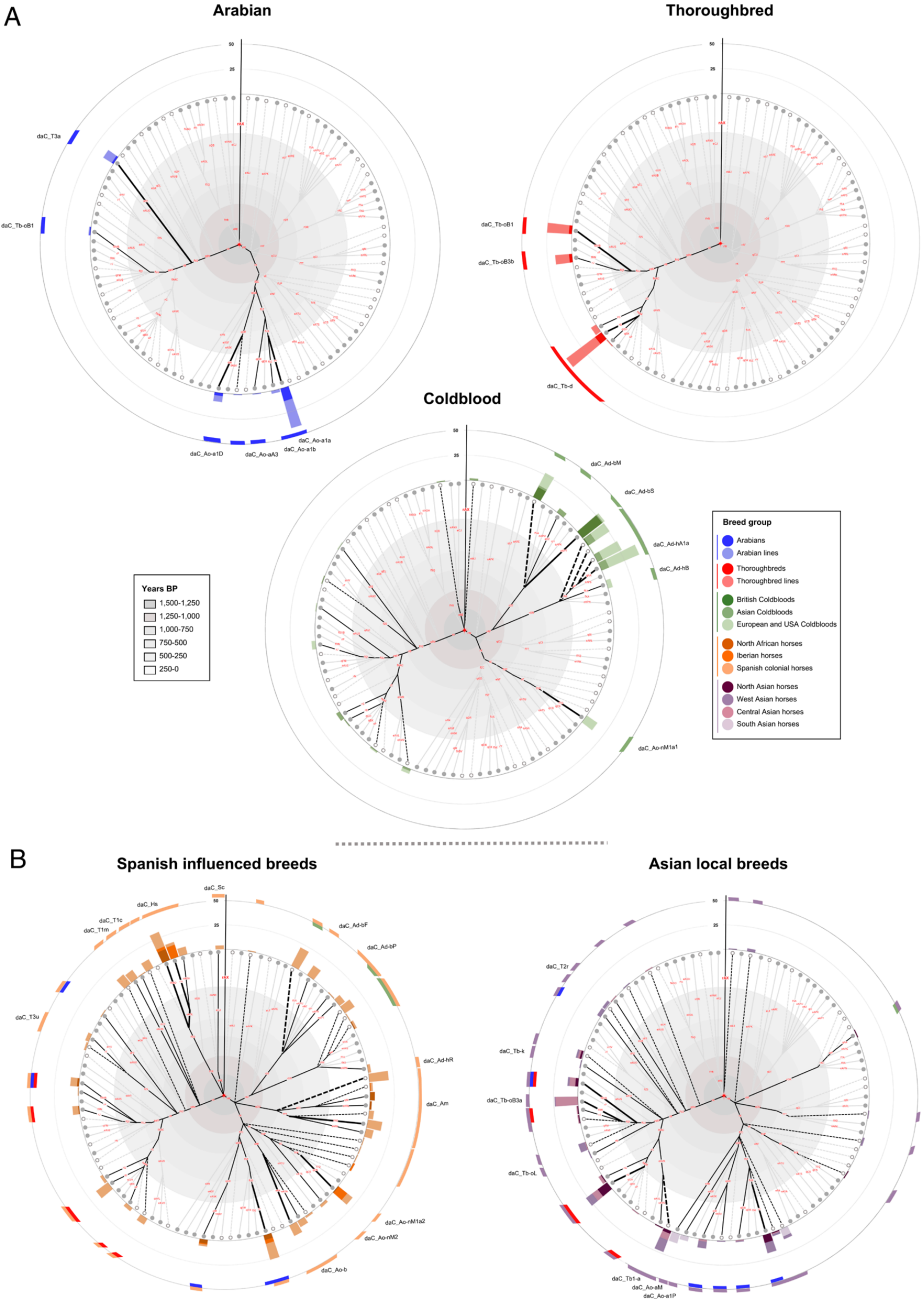
MSY HT spectra in breed groups. Sunburst plots showing the frequency of HTs in different groups of breeds (details in Dataset S1). The HT topology of the Crown (details in *SI Appendix*, Fig. S4), with branching points approximately scaled to the time frames of emergence (Fig. 1*B* and *SI Appendix*, Table S1), is given in the center. HT-DV are shown in red. *HTs allocated on internal nodes after genotyping are marked with dashed lines that originate from the corresponding internal node. Colored shadings on the outer circle accentuate the HTs in each group and branches connecting the HTs in a group are bold. (*A*) Discrete HT signatures in Arabian (n = 70), Thoroughbred (n = 93), and Coldblood (n = 214) groups. (*B*) MSY HT spectra of horses with Spanish influence (n = 345) and local Asian breeds (n = 194).

The MSY HT spectrum revealed the dominance of a few very frequent HTs in the Arabian, the Thoroughbred, and the Coldblood groups (Fig. 2*A*). The trend was also evident from the lower haplotype diversity (Hd) levels in the Arabian, Thoroughbred, and Coldblood groups, while the horses of the Spanish-influenced group exhibited the highest Hd with a value of 0.961, followed by local riding and light draft breeds from Europe and the United States (0.953) and local riding and light draft breeds from Asia (0.921, see Table 1).

**Table 1.** Descriptive statistics of MSY HTs in main breed groups, including Hd and SD

Breed group	Number of samples			Number of HTs			Hd (SD)	
	Σ	daC	Non daC	Σ	daC	Non daC	daC	daC and Non daC
Arabian	69	69	–	7	7	–	0.816 (0.039)	–
Thoroughbred	93	93	–	5	5	–	0.874 (0.039)	–
Coldblood	214	195	19	21	18	3	0.884 (0.009)	0.90 (0.008)
Spanish-influenced breeds	345	345	–	45	45	–	0.961 (0.002)	–
Local riding and light draft-Asia	210	194	16	37	35	2	0.921 (0.009)	0.928 (0.056)
Local riding and light draft-Europe, USA and other colonial territories	440	323	117	53	47	6	0.953 (0.005)	0.960 (0.003)
European and USA riding breeds	146	146	–	22	22	–	0.911 (0.012)	–
**Total**	1,517	1,365	152	102	93	9	0.962 (0.002)	0.966 (0.002)

We interpret the circumscribed HT pattern in the Arabians, Thoroughbreds, and Coldbloods as a hallmark of the pronounced line breeding, which focuses on a small number of commonly used stallions. We estimated that the highly frequent MSY HTs in these breeds emerged between 250 and a maximum of 600 y BP (Fig. 2*A*), which supports the idea of the recent amplification of the lineages.

Descendants of Arabian and Thoroughbred foundation sires grouped in accordance with previous studies (6, 30, 32) mainly into daC_Ao-a and daC_Tb (details including HTs are given in *SI Appendix*, Table S2 and Dataset S1).We define discrete Crown MSY signatures in Coldbloods (Fig. 2*A*). In our Crown dataset of 195 Coldbloods, HTs in the ~280-y-old subHG (sHG) daC_Ad-h were the most common (51.3%), followed by HTs in the ~435-y-old sHG daC_Ad-b (30.7%) and daC_Ao-nM1a1 (6.1%). The remaining 28 Coldbloods (14.4%) were located all over the Crown, including nine inner node “*HTs” (information on breed, sire line, and HT is given in Dataset S1). We also observed HTs in the daC_Tb-d sHG, indicating undocumented Thoroughbred influence in Coldbloods (Fig. 2*A*). The differences in HT composition clearly mirror geographic separation and regional breeding strategies in Coldbloods. Breeds from the British Islands had almost exclusively sHG daC_Ad-b, whereas the Coldbloods from Europe and north America mainly grouped into sHG daC_Ad-h and few males carried daC_Ad-b HGs or daC_Ao-nM.

Overall, our analysis confirmed the circumscribed MSY-HT signatures due to recent selective breeding in Arabians and Thoroughbreds and we detected a comparably distinct pattern in Coldbloods.

### The Spanish Dissemination of the Crown.

Having pinpointed the discrete patterns of recent line breeding in Arabians, Thoroughbreds, and Coldbloods, we explored other influences that shaped the Crown HG. In particular, we wondered whether the dissemination of stallions through the ancient geographical “melting pot,” the Iberian Peninsula, from the occupation of the Moors, the Reconquista, and the colonialization of the American continent, was a major force in the propagation of the Crown. Due to the lack of recombination, MSY HTs retain information on the accumulation of genetic variation and allow ancient dispersal to be separated from subsequent overlying recent introgressions (26, 40).

**Fig. 3.**
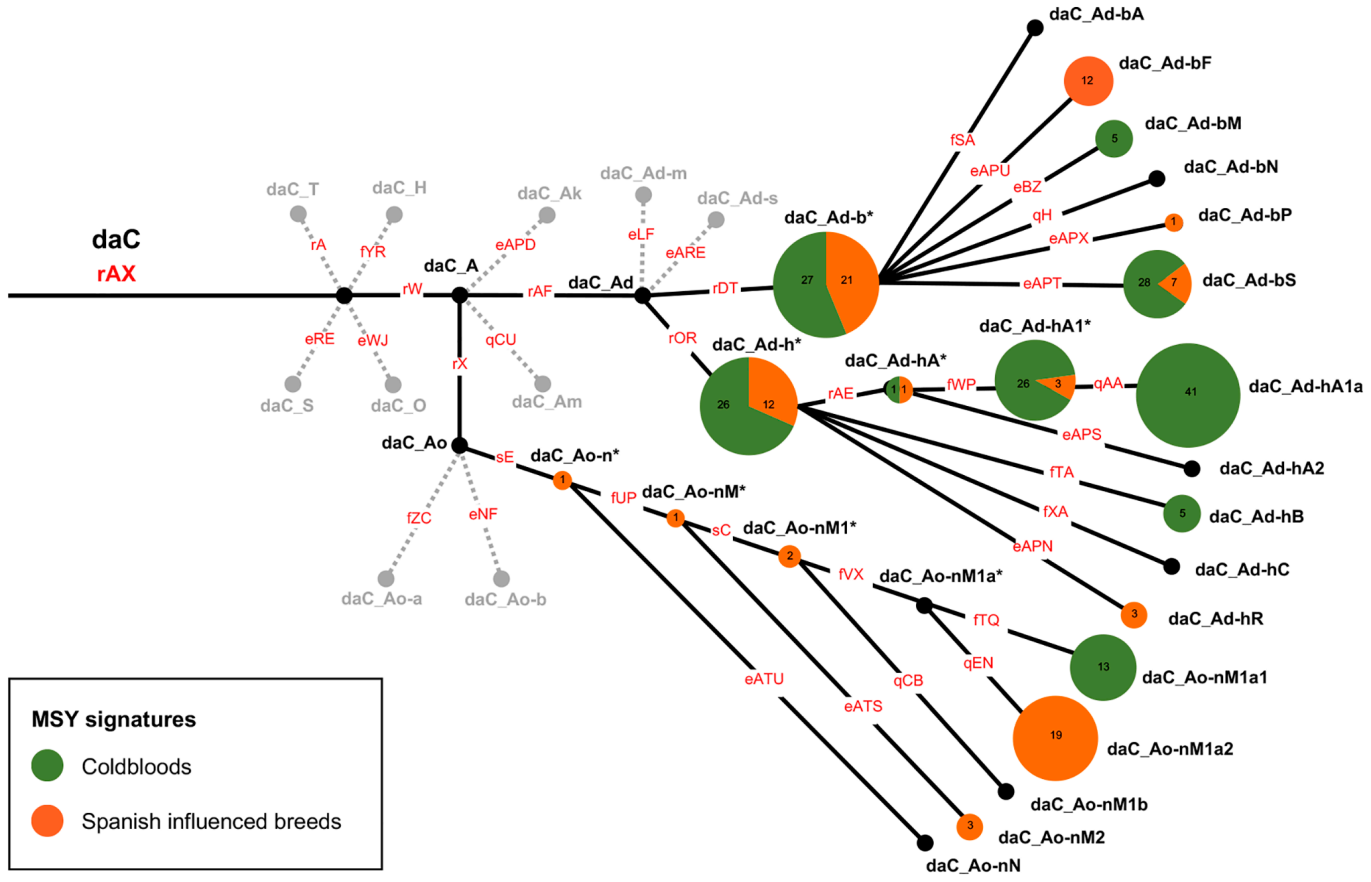
MSY signatures in sHGs daC_Ad-b, daC_Ad-h, and daC-Ao-nM in Coldbloods and Spanish-influenced breeds. HTs are represented as pies, scaled to the number of samples with distinctive HTs (indicated numerically for each pie portion). *HTs are placed on the branching points, while HTs that were not detected in the sample set are shown in black. Lines and variant names in red correspond to the HT topology in *SI Appendix*, Fig. S4, HGs in gray are collapsed.

Because of the broad and early onset of this “Spanish dissemination,” we expected the Crown MSY traces to be more diffuse than those of Arabians, Thoroughbreds, and Coldbloods. We examined MSY HTs in the group of breeds with recorded Spanish influence, composed of 345 horses of 40 breeds, including 33 North African, 35 Iberian, and 277 Colonial Spanish horses (Dataset S1). The horses showed a broad spectrum of diversified lineages (Figs. 2*A* and 3*B* and Table 1) and included 45 HTs dispersed over 23 mostly early Crown sHGs (MRCA 750 to 500 y BP). Arabian, Thoroughbred, and Coldblood signatures showed up in 80 individuals, whereas 18 defined and 17 *HTs were unique to the Spanish-influenced group. We detected three private and predominantly Spanish sHGs (daC_Am, daC_Ao-b, daC_Hs) along with HTs in the Coldblood sHGs (daC_Ad-b, daC_Ad-h, daC_Ao-nM) and some early branching HTs in major HG daC_T (daC_T1m/c and daC_T3u). sHGs daC_Am and Ao-b were the most abundant in Colonial Spanish breeds (Dataset S1), while daC_Hs occurred at similar frequencies in Colonial Spanish and Iberian breeds, North African Barb, and the Canadian Ojibwe horse (former called Lac La Croix Indian Pony). The multiple early branching *HTs (e.g., daC_Am*, daC_Ao*, etc.) indicate the lack of many private Spanish MSY lineages in the current MSY phylogeny.

The broad spectrum of MSY HTs in Spanish influenced breeds is consistent with the massive propagation of stallions through ancient Spain in the past 700 y, as narratively suggested (11).

Our data confirm that the Spanish dissemination was an early driver of the predominance of the Crown—toward the American continent and into Europe. Interestingly, the three Coldblood signatures are surrounded by HTs detected in the Spanish group (Fig. 3). The HTs in the Spanish group branch basally (in sHG daC_Ad-b, daC_Ad-h, and daC_Ao-nM), while Coldbloods predominantly carry terminal HTs, providing a clear indication of a Spanish origin of the paternal lineages dominant in today’s Coldbloods.

### Significant Propagation of the Crown through Horses from Western Asia.

Recent breeding and the earlier Spanish dissemination are not sufficient to explain the Crown HT spectrum. In particular, the origin of the HGs daC_Tb and daC_Ao-a that harbor the ancestors of Thoroughbreds and Arabians was unclear. Given the impact of Asian Oriental Horses in history (5, 6, 8), we compiled a panel of 26 local breeds mainly from western Asia (n = 194 samples in daC, breeds listed in Dataset S1). A remarkably high proportion (80 males/41.2%) carried HTs indicative of recent undocumented breeding with Thoroughbreds, Arabians, and Coldbloods. Of the remaining 114 horses, 99 (86.4%) grouped into the HGs daC_Tb or daC_Ao-a, with the other fifteen clustered into HG daC_T and daC_A forming basal *HTs (see Fig. 2*B* and Dataset S1 for details). Asian local horses, thus, provide the missing piece of the puzzle, representing a broad spectrum of HTs in daC_Tb (MRCA 635 ± 148 y BP CI) and daC_Ao-a (MRCA 781 ± 181 y BP CI) HGs. While Thoroughbreds and Arabians spread daC_Tb and daC_Ao-a HTs across the globe, sister HTs evolved and are nowadays found in local Asian breeds. For example, sHG daC_Tb-oB (MRCA 377 ± 115 y BP), which comprised the HTs of two Thoroughbred and several Arabian sires, is highly frequent in 24 Turkoman lineage horses via HT daC_Tb-oB3a.

The emergence of numerous sHG within dac_Tb and daC_Ao-a in local Asian horses in the past 500 to 600 y suggests a second dissemination (later than the Spanish distribution) centered in west Asia. We suspect that this spread of horses, which we refer to as the “West Asian dissemination,” took place via the trade routes to the east and north in the context of the Ottoman Empire. The West Asian dissemination is sufficient to account for the origin of the founders of Thoroughbreds and the majority of Arabians.

### MSY HTs as Ancestry Predictors—Inferring the Paternal History of Local Horses and Ponies.

**Fig. 4.**
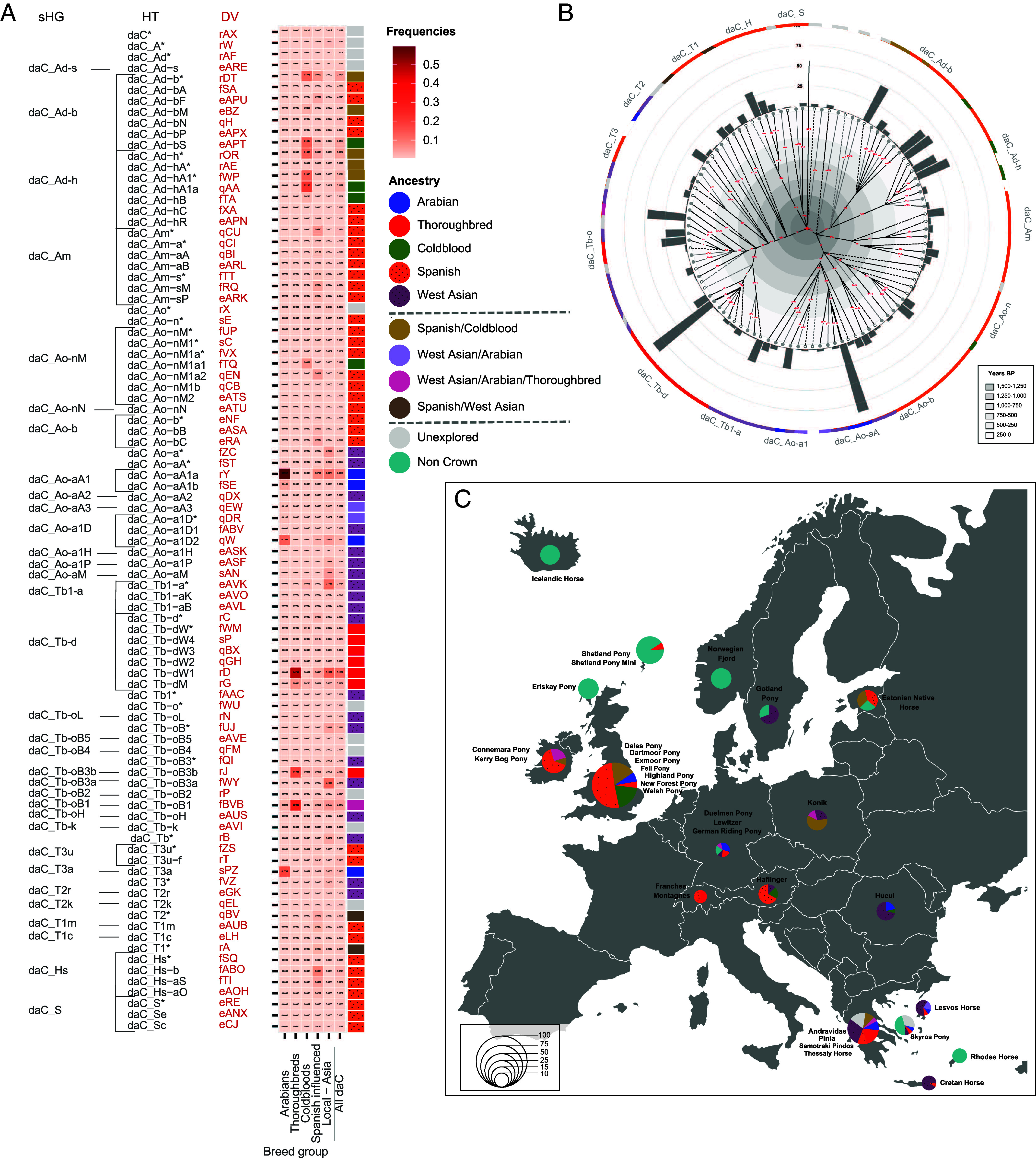
MSY paternal ancestry prediction. (*A*) All Crown (daC) HTs detected in our dataset with HT-DV denoted. HT frequencies in breed groups are shown as a heatmap (values also indicated numerically in each tile). Rectangles on the right indicate corroborated ancestry signature for each HT, colored according to the legend. Details on ancestry signatures are given in *SI Appendix*, Table S2. (*B*) Joint MSY HT distribution of 896 individuals from the five main breed groups in Fig. 2. Topology and HT-DV (red) are shown in the center (detail in *SI Appendix*, Fig. S4 and Dataset S2) and dashed lines trace to *HTs. The inner gray spectrum illustrates estimated branching times (*SI Appendix*, Table S1). Ancestry signatures are given in the outer circle. (*C*) Paternal ancestry inference in 34 European local riding, light draft horse, and pony breeds (341 males). Pies are scaled to the number of individuals and colored according to the paternal ancestry. The full information on breeds and their HTs is given in Dataset S1.

The three recent breeding influences (Thoroughbred, Arabian, Coldblood) and the two earlier expansion events (Spanish and West Asian) almost completely explain the origin of Crown HTs. The signatures of MSY HTs can now be used as a proxy to infer the paternal ancestry of populations. Based on HT frequencies in our defined breed groups (Fig. 4*A*), on narrative history, on sire line information, and on HT topology (details in *SI Appendix*, Table S2), we defined 72 HTs, including basal *HTs, as unique predictors of a certain ancestry. Four HTs indicate Arabian, seven Thoroughbred, four Coldblood, 37 Spanish, and 20 West Asian ancestries. Ten Crown HTs give varied ancestry signals, as they were detected in several breed groups (e.g., daC_Ad-h* in Spanish/Coldblood), while the origin of nine Crown HTs (mostly inner node clustering) remains unexplained (Fig. 4*A* and *SI Appendix*, Table S2). It is important to note that although we built our analysis on the best resolved MSY topology to date (7), the Crown HT spectrum is still underestimated. Especially in South American, and East Asian sHGs, we detected several basal *HTs and ancestry prediction is therefore less detailed in those groups.

To test the potential for analysis of paternal ancestry, we considered 48 breeds including regional riding and light draft horses and ponies from Europe, the United States, and other colonial territories (details in Dataset S1). Pedigree documentation dated back only a few generations or was unavailable, so the origin of the breeding stallions was largely based on narrative history. We genotyped 440 males, of which 92 (20.9%) showed recent influences of Thoroughbreds (n = 48), Arabians (n = 20), or Coldbloods (n = 24); 152 (34.5%) showed indications of early disseminations (97 Spanish, 55 West Asian) and 67 (15.2%) carried a varied ancestry signal. Only twelve animals carried Crown HTs that we could not explain. Standardbred (daC_Tb-dM) and Thoroughbred lineages derived from the stallion “Whalebone” (daC_Tb-dW1) (30) dominated in local US breeds (see Dataset S1 for details), while we detected non-Crown HTs in 117 males from northern Europe, Greek Islands, and north America. The findings agree with the results of previous studies (6, 7, 21) (for details, see Dataset S1). We need to mention, that we faced a difficulty of distinguishing the many recently established patrilines in modern breeds (e.g., in HT Tb-oB1 or Ad-hA1a). Here, the resolution could be improved by implementing faster evolving MSY short tandem repeat markers (26).

The composition of paternal ancestry of 34 European local riding and light draft horse breeds (a total of 341 horses) showed pronounced regional differences (Fig. 4*C*). We detected the Arabian signature in 18 horses of 10 local European breeds (see Dataset S1 for details) and found the Arabian HT daC_Ao-aA1 preferentially in horses from the United Kingdom and Germany, daC_Ao-a1D2 largely in Greek breeds (Lesvos, Andravidas) and daC_T3a in horses from Romania (Hucul horse breed) and Greece (Pinia). We found a broad Arabian signature with three Arabian HTs (daC_Ao-aA1a, daC_Ao-a1D2, and daC_T3a) in British New Forest Ponies (n = 4). We noted ancestry signatures of Thoroughbreds in 21 males of twelve breeds. The pronounced Arabian and Thoroughbred signatures are not surprising considering the reported use of these stallions to improve local stocks (3). We also observed apparent Coldblood ancestry and 35 horses (of 10 breeds) carried the varied Spanish/Coldblood signature. Most notably, the Spanish-derived sHG daC_Ad-b, including the typical British Coldblood HT Ad-bS, is predominant in six British and Irish pony breeds (Connemara Pony, Dales Pony, Dartmoor Pony, Fell Pony, Highland Pony, and Kerry Bog Pony). This finding supports the close relationship among British pony breeds (41), the connection between British breeds of different types (39, 42), and the close relatedness of Spanish horses and British ponies (43). We did not detect sHG daC_Ad-b in British Island breeds such as the Shetland and the Eriskay Pony or in the Exmoor Pony, consistent with their isolated breeding history.

We deduced a West Asian ancestry for 54 males across six geographic regions, mainly in south-eastern parts of Europe (ponies from Greek Islands and the Romanian Hucul breed) but also in nine Swedish Gotland Ponies, which could be attributed to a single recent sire, as strong inbreeding in this breed (44) results in the dominance of a single patriline. MSY lineage tracing in local populations revealed the impact of the Spanish and West Asian dissemination in addition to the more recent influence of Arabians, Thoroughbreds, and Coldbloods, as well as highlighting regional differences and similarities between breeds.

## Discussion

The historical evolution of domestic horses is determined by a recurring pattern of phenotypic adaptations to ever-changing human needs and aspirations. Complex waves of migration and breeding strategies in the last millennium have meant that a handful of influential sires have given rise to the modern horse population (11, 27, 30). We have used MSY data to decipher the key factors that led to the worldwide dominance of Oriental bloodlines and to test the myths surrounding the origins and legacy of individual breeding stallions (3, 10).

Our dataset of 1,517 individuals from 189 distinct breeds accentuated three recent breeding influences (Thoroughbred, Arabian, and Coldblood). The discrete MSY signatures of Thoroughbreds and Arabians can be explained by the strong tradition of selective breeding (3, 45). Likewise, the abundance of their HTs in other breeds stems from the excessive use of Arabian and Thoroughbred patrilines to shape phenotypes in the recent past (6, 9, 30, 32). This is best illustrated by European and US riding horses, which contain almost exclusively Thoroughbred and Arabian HTs (*SI Appendix*, Fig. S6), but the impact is also evident in local breeds in Europe (Fig. 4*C*) and other regions of the world (Fig. 2*B*). We also report a similarly circumscribed MSY signature in Coldblood horses with their distinct HTs in daC_Ad-h/Ad-b/Ao-nM sHGs (Figs. 2 and 4*A*). The clear Coldblood imprints result from the intensive and organized breeding of working horses in the 19th century, which was followed by drastic population declines (3, 46).

We delineate two routes of dissemination of Crown lineages before recent intentional breeding: the spread of Oriental stallions through the Iberian Peninsula to Europe and the New World in the 8th to 16th century and a slightly later dispersal of stallions from West Asia. A wide spectrum of Crown HTs was dispersed via ancient Spain, as expected from the long and geographically broad dissemination of horses via the Iberian Peninsula. The diverse MSY HTs in Spanish-influenced breeds are in line with other genomic regions, such as autosomal and mtDNA (19, 47). However, the observation of Crown lineages in these breeds proves their oriental ancestry, gives no evidence of an ancient Iberian lineage (5), and is in concordance with previous findings (21) of Crown HTs in modern and historical American horses. MSY HTs support the distinctiveness and Spanish origin of New World breeds (11, 12, 16, 19, 48, 49) and reveal the exchange of horses from north Africa and the Iberian Peninsula at several periods (cf ref. 33). Our results confirm the Spanish origin of the HTs in todays’ Coldbloods and British Ponies (Fig. 4 and Dataset S1). The close genetic relationship between Spanish horses and Coldbloods (21) as well as with European ponies (19, 50) was clear before our study; our hierarchical MSY topology shows the direction of genetic transmission.

**Fig. 5.**
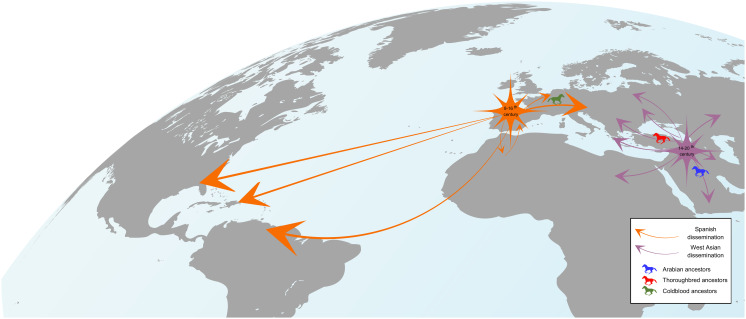
Schematic illustration of the spread of Oriental Horses. The earliest Spanish dissemination, from which the Coldblood lineages arose, is indicated with orange arrows, the subsequent West Asian dissemination, from which the Arabian and Thoroughbred lines emerged, in purple. Approximate time periods of each dissemination are indicated with a star.

The second wide dissemination of Oriental Horses was deduced from the MSY HT spectrum detected in local horse breeds, especially from south-west Asia, another historically important region. These horses largely carry daC Tb and Ao HTs, which are not found in Thoroughbreds and Arabians (Fig. 2*B*). Our fine-scaled MSY genotyping reveals these “non-Thoroughbred Tb” and “non-Arabian Ao” HTs widely distributed and not geographically separated. While previous MSY studies attributed the daC_Tb HG to a Turkoman origin (6) and HTs in daC_Ao mainly to Arabian horses (32), our results show a more differentiated picture. Central Asian breeds, such as the Akhal Teke, Turkoman, Kurd, and Caspian horse share a recent common ancestor with Thoroughbreds and Arabians on the MSY tree (Fig. 2*B*), consistent with autosomal and mtDNA findings (14, 39, 42). The most parsimonious explanation is that a second major expansion drove the dissemination of Oriental Horses from Asia, forming the source of the Arabian and Thoroughbred sires that became so influential later (Fig. 5).

Our interpretation is that the Spanish dissemination fueled the initial spread of Crown lineages to central Europe and the New World (from the 8th to the 16th century), while the second dissemination originated in central/western Asia, including the Arabian Peninsula, brought Oriental Horses into Europe, north Africa, and the rest of Asia. The two expansions overlapped in time (8, 12, 13), as reflected in the estimates of the timing of the MSY HGs (*SI Appendix*, Table S1). The West Asian dissemination can be linked to the spread of the Ottoman Empire (from roughly the 14th to the 20th century) (13, 51). There are many possible explanations of the gene flow of west Asian horses, as horses were very valuable in the Ottoman Empire, being used for warfare, agriculture, transportation, and hunting, as well as being a status symbol and a frequent gift (8). There were also intensive selection strategies (52), which might have driven the overrepresentation of particular HTs in daC_Ao and daC_Tb (e.g., daC_Tb1-a), as well as in Turkoman, Arabian, and Thoroughbred patrilines. The West Asian dissemination spread Crown HTs far from Europe and we detected HTs unique to South Asian horses such as Pakistan horses in daC_Ao-a1P and Marwari horses in daC_Ao-aM, which are most probably signatures of central Asian horses that were transmitted eastward (8, 13). We should also acknowledge that the MSY is prone to strong drift effects (53) and the continuous selection on stallions has accelerated the reduction of variability (5). Many MSY lineages have become extinct in the recent past and contemporary populations cannot act as direct proxies for all historic migrations. A complete account of the spatiotemporal trajectories of HTs will require the integration of historical samples (54) in the future.

Our study enables a meaningful genetic evaluation of the paternal ancestry of any modern horse breed. We have defined a classification scheme and tested it on local riding and light draft horses and ponies. The results showed a strong impact of Arabian, Thoroughbred, and Coldblood lineages, in line with the grouping obtained from autosomal markers in those breeds (38, 39, 42, 55). We also found an MSY HT pattern similar to that of British Ponies in Australian Waler horses (Dataset S1) and in feral Kaimanawa Horses (36), presumably resulting from the British colonization of Australia and New Zealand (56). This finding prompted us to review the signals of colonization events and earlier migrations. We found signatures of the West Asian dissemination in Greece, as a consequence of the Mediterranean passage from Asia to Europe, as well as in Romania and Poland, corresponding to the history of territories influenced by the Ottoman Empire (8, 13). We also identified distinctive signatures of central Asian horses transmitted eastward, for example, in Pakistan horses and Marwari horses. Although little attention has been paid to east Asian populations, we expect them to be a conglomerate of autochthonous variation (i.e., non-Crown HTs) and historical influences, as is the case for Mongolian populations (34, 35). It would be interesting to investigate Asian populations and interpret the MSY patterns within the framework of our ancestry signatures. Furthermore, we focused here on lineages of the Crown group; while the non-Crown HTs in modern and local breeds require further investigation.

Here, we have demonstrated the power of the MSY to uncover the complex recent history of modern horse breeds. By tracing the legacy of Oriental stallions, we have demonstrated the inseparability of horse and human history, deciphered former unknown connections between geographically and phenotypically different horse breeds, and highlighted the consequences of intensive animal breeding. Our work opens a different level of opportunity to capture the historical development of breeding populations and serves as a meaningful decision-making aid for horse breeding management and conservation priorities.

## Materials and Methods

### Ethics Statement.

The study was approved by the institutional ethics and welfare committee of the University of Veterinary Medicine Vienna and executed according to the Good Scientific Practice guidelines and national legislation (ETK-10/05/2016). Biological material of horses was provided by private horse owners, or obtained from breeding associations and collaborators in compliance with the animal welfare standards in the country in question. The blood samples of Lipizzan horses were collected ~25 y ago within the framework of the EU Kopernikus project “Biotechnical methods in the maintenance of the genetic diversity in the Lipizzan horse breed” (Project No. IC15CT96-0904). Permission for the scientific use of the samples was granted by all horse owners, stud farms, and collaborators. Sample encoding ensured the anonymity of individuals included in the study.

### Estimation of the Divergence Times within the Crown HG.

To estimate divergence times within the Crown, we constructed a Bayesian tree by utilizing a fasta file containing 170 horse samples and 2,678 high-quality SNPs (out of 2,966 published) from a previous study (7). The substitution model was chosen according to datamonkey (57). We generated an xml file using BEAUTI version 2.7.3 (58) with the following parameters: a gamma site model and HKY model with the proportion of invariant sites (0.9995), horse Y mutation rate 1.69^−9^ mutations per site per year (30) estimated based on a generation interval of 10 y, upper mutation rate 2.11^−9^, and lower mutation rate 1.41^−9^ mutations per site per year. The Constant Population Model was selected and we used the Strict Clock model as the branch rate model. To ensure accurate estimation, 20,000,000 MCMC runs were generated using BEAST, and two independent runs were performed. The output trees were combined with TreeAnnotator and the consensus tree was visualized with FigTree 1.4.4 (https://github.com/rambaut/figtree/). The ESS value indicating robustness of the final tree (ESS = 5243.1) was obtained with Tracer v1.7.2 (59). For comprehensive visualization, we placed the divergence times of the main nodes within the Crown estimated with the Bayesian approach (95% Highest Posterior Density) and the corresponding CI (full information in *SI Appendix*, Table S1) on a maximum parsimony tree constructed from 512 variants and 121 samples (Dataset S3, data derived from ref. 7) with ape 5.7-1 (60) and phangorn 2.11.1 (61) R packages.

### The Sampling.

We constructed a sample set containing 1,517 individuals of 189 horse breeds. The breeds originated from 60 countries/regions worldwide (Dataset S1). We selected a maximum of 15 individuals per breed (*SI Appendix*, Fig. S1). If available, we considered the documentation of the horses’ male ancestry, from studbooks and pedigree records, to cover all and avoid overrepresentation of sire lines in a breed with the so‐called “founder sampling” (62). First, we reconstructed paternal genealogies of focal breeds following ref. 32. The reconstructed male-tail lineages were stored as a string containing the names, birth years, birthplaces, and breed affiliations of ancestors to the greatest extent possible. Based on this information, we selected a representative collection of foundation sires in a breed and their sublines. In cases where more than 15 sire lines were represented in a breed, one individual per patriline was randomly selected. If fewer than 15 sire lines per breed were reported, we avoided bias toward certain sire lines by randomly taking a maximum of three individuals per stallion line (most recent foundation sire). Paternal lineage information is provided for each sample when available in Dataset S1.

Sufficient male-tail line information was available for 507 males and absent for 1,010 individuals. For breeds with patriline information represented in fewer than 15 individuals, we randomly selected stallions without patriline information until we reached the threshold of 15 horses in the breed. Independent sampling collections were considered in the sample set construction, when feasible. Some breeds are represented with a limited number of samples (n < 5) but were included for the sake of completeness. We divided the sample set into 13 “geographic groups” based on the information on the country/region of the breeds’ origin (following refs. 3, 7, 28, and 38) and extensive literature research: Africa (n = 36), north Africa (n = 33), north Europe (n = 323), central and western Europe (n = 280), south and eastern Europe (n = 102), the Iberian Peninsula (n = 45), Latin America (n = 145), north America (n = 258), north Asia (n = 94), central Asia (n = 87), west Asia (n = 80), south Asia (n = 25), and other colonial territories (n = 9) (*SI Appendix*, Figs. S1 and S2).

We also classified the horses according to their narrative and documented breed history, male-tail line information, and type into “breed groups” following refs. 3 and 38. We formed main groups with documented recent directional breeding: the Arabian group (including Arabians and Arabian lines in other breeds) (n = 69), the Thoroughbred group (including Thoroughbreds and Thoroughbred lines in other breeds) (n = 93), and the Coldblood group (heavy horses, n = 214). We grouped North African, Iberian, and Colonial Spanish horses as the “Spanish-influenced group” (n = 345). “Riding Europe and US horses” (n = 146) formed the fifth group, and the remaining 75 local breeds of various types with unstudied progenitors made the group of “local riding and light draft horses from Europe, the United States, and other colonial territories” (48 breeds; n = 440) and the group “local riding and light draft horses from Asia” (27 breeds; n = 210) (*SI Appendix*, Fig. S1 and Dataset S1). For detailed analysis, the main groups were further divided by geographic region.

### DNA Extraction.

Genomic DNA was obtained from collaborators or by extraction from hair roots, blood, or semen using nexttec™ DNA Isolation Systems (Hilgertshausen, Germany). DNA was diluted to a concentration of 5 ng/μL with TE buffer. Detailed information on biosamples is listed in Dataset S1.

### MSY Genotyping.

We created a downscaled HT structure of the most recent horse Y phylogeny (7) (as schematized in *SI Appendix*, Fig. S3). We chose 124 HT-determining “key” variants (118 SNPs, five short Indels, and one microsatellite, see *SI Appendix*, Fig. S4 and Dataset S2) that determine 66 terminal and 35 inner nodes in the Crown and 17 terminal HTs and five inner nodes outside the Crown (*SI Appendix*, Fig. S4). This structure served as a genotyping backbone. The Crown HTs were condensed into 38 so-called sHGs for easier manipulation and visualization (*SI Appendix*, Fig. S4 and Dataset S1).

We determined the allelic states of variants by means of competitive allele-specific PCR genotyping assays (KASP™, lgcgroup.com) on a CFX96 Touch® BioRad Real-Time PCR machine, using the standard KASP™ genotyping protocol (lgcgroup.com). Samples with known allelic states served as positive controls, while DNA from females and nontemplate controls were included as negative controls. Raw data were analyzed and visualized via Cartesian plots with Bio-Rad CFX Manager 3.1® software (BioRad). For 63 individuals, the tetranucleotide microsatellite fBVB (GATA14/GATA15) was genotyped following the protocol given in refs. 30 and 32. Information on screened variants is available in Dataset S2.

Genotyping was carried out in a successive manner (*SI Appendix*, Fig. S3). The sample was first tested for the Crown-determining variant rAX. If it carried the derived allele for rAX (rAX_C), it was tested further, following the hierarchy of the Crown backbone until the terminal branches. We considered the reported HT frequency in the breed under investigation in the genotyping process, e.g., we first tested an Arabian horse for variants rW (determining HG daC_A), rY (daC_Ao-aA1a), and qW (daC_Ao-aD2), based on the information in ref. 32. If a sample carried the ancestral allele for variant rAX, we tested it for non-Crown variants as described above.

### Data Analysis.

MSY HTs from 1,507 horses were determined with KASP genotyping, while HTs of 10 individuals were obtained from published information (7, 32) The genotyping of 124 variants of 1,517 samples was merged into a single file. We imputed the allelic states of markers that we did not test according to the published HT structure (7) and encoded HTs into a matrix of variant allelic states (Ref/Alt-0/1) for each individual (*SI Appendix*, Fig. S3 and Dataset S2). HT frequencies were calculated by direct counting, while Hd was calculated after (63) with R packages pegas (64) and adegenet (65). Data manipulation and descriptive statistics were performed with RStudio 4.2.2. (66) and the tidyverse collection of packages (67). We generated the MSY HT topology as a median-joining HT network with the Network 10.2 (68). The output was redrawn as a HT frequency plot for each breed group with RStudio (66) and Canva Pro (Canva, https://www.canva.com/pro/, last accessed 30 August 2024).

### Informed Consent Statement.

For all biosamples used in the study, we acquired a written consent for scientific use from horse owners and breeders, or collaborators.

## Supplementary Material

Appendix 01 (PDF)

Dataset S01 (XLSX)

Dataset S02 (XLSX)

Dataset S03 (XLSX)

## Data Availability

Codes are available on GitHub (https://github.com/larad1010/horseYancestree) (69).
