# Supplementary material for: The global spread of Oriental Horses in the past 1,500 years through the lens of the Y chromosome
Source: Proc Natl Acad Sci U S A. 2024 Nov 18;121(49):e2414408121. doi: 10.1073/pnas.2414408121 (PMC11626155; doi:10.1073/pnas.2414408121)
Supplement: Supplementary file 1 — Appendix 01 (PDF) [file pnas.2414408121.sapp.pdf]

PNAS

Supporting Information for

The global spread of Oriental Horses in the past 1,500 years through the lens of the Y chromosome

Lara Radovic[1,2], Viktoria Remer[1], Doris Rigler[1], Elif Bozlak[1,2], Lucy Allen[1], Gottfried Brem[1], Monika Reissman[3], Gudrun Brockmann[3], Katarzyna Ropka-Molik[4], Monika Stefaniuk-Szmukier[4], Liliya Kalinkova[5], Valery V. Kalashnikov[5], Alexander M. Zaitev[5], Terje Raudsepp[6], Caitlin Castaneda[6], Ines von Butler-Wemken[7], Laura Patterson Rosa[8], Samantha Brooks[9], Miguel Novoa-Bravo[10], Nikos Kostaras[11], Abdugani Abdurasulov[12], Douglas F. Antczak[13], Donald C. Miller[13], Maria Susana Lopes[14], Artur da Câmara Machado[14], Gabriella Lindgren[15], Rytis Juras[16], Gus Cothran[16], Barbara Wallner[1,*]

*Barbara Wallner
Email: barbara.wallner@vetmeduni.ac.at

This PDF file includes:

Supporting Figures S1 to S6
Supporting Tables S1 and S2
Legends for Datasets S1 to S3
SI References

Other supporting materials for this manuscript include the following:

Datasets S1 to S3

Supporting Figures

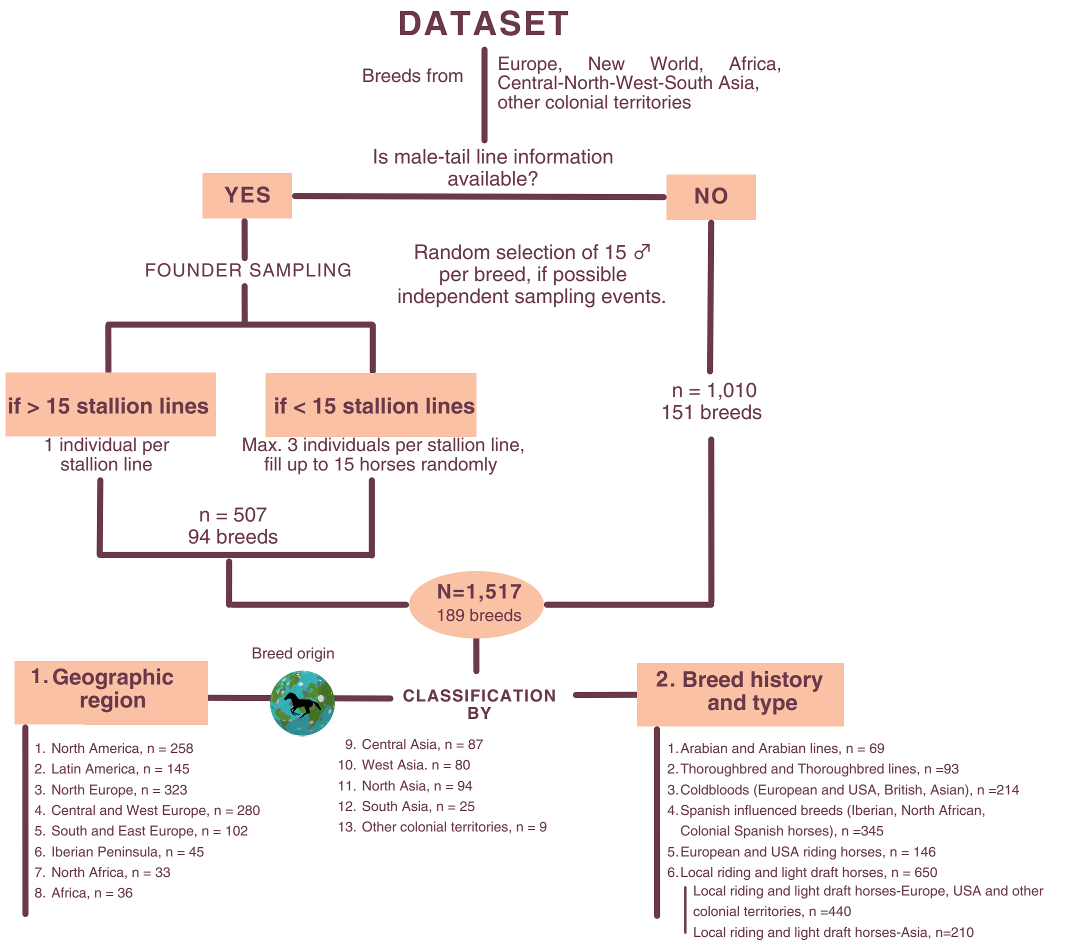

S1 Fig. Construction of the dataset. Workflow describes the design of the dataset (n=1,517) used in the study. In the sampling process, breed overrepresentation was averted by taking a maximum of 15 individuals per breed. Reconstruction of the male tail line information was carried out as described by (1) to avoid bias towards certain sire lines in the dataset ('founder sampling', (2)). Based on pedigree information and breed ancestry obtained from literature research, samples were assigned to different geographic groups according to their origin, as well as breed groups describing major breeding influence that affected their breed.

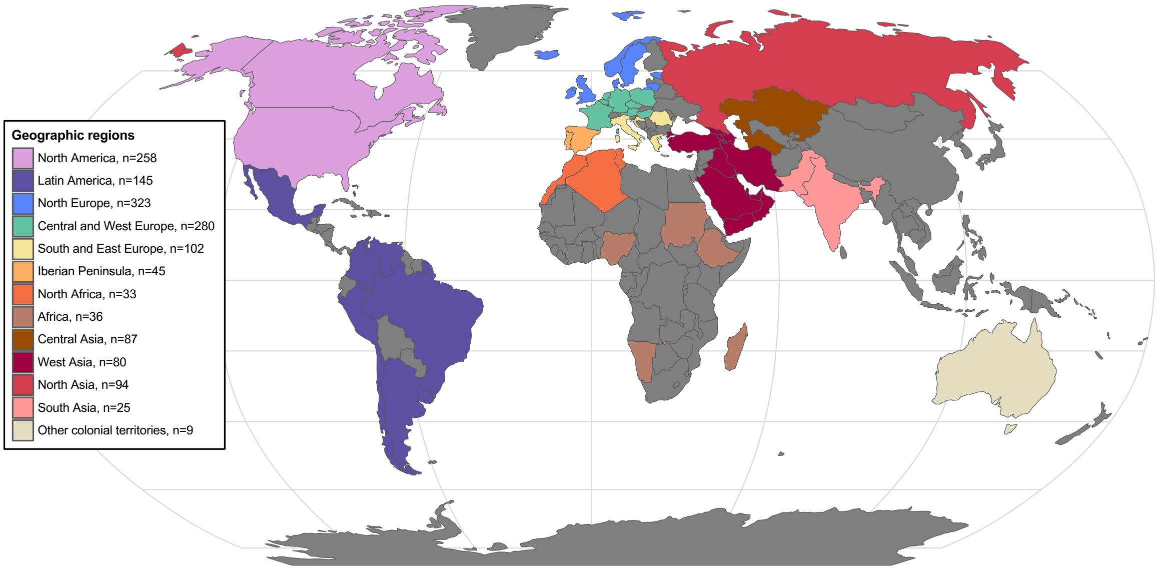

S2 Fig. Distribution of geographic regions. World map shows clustering of 1,517 samples used in the study into 13 geographic groups, denoted with different colors, comprising 60 countries/regions of the breeds´ origin.

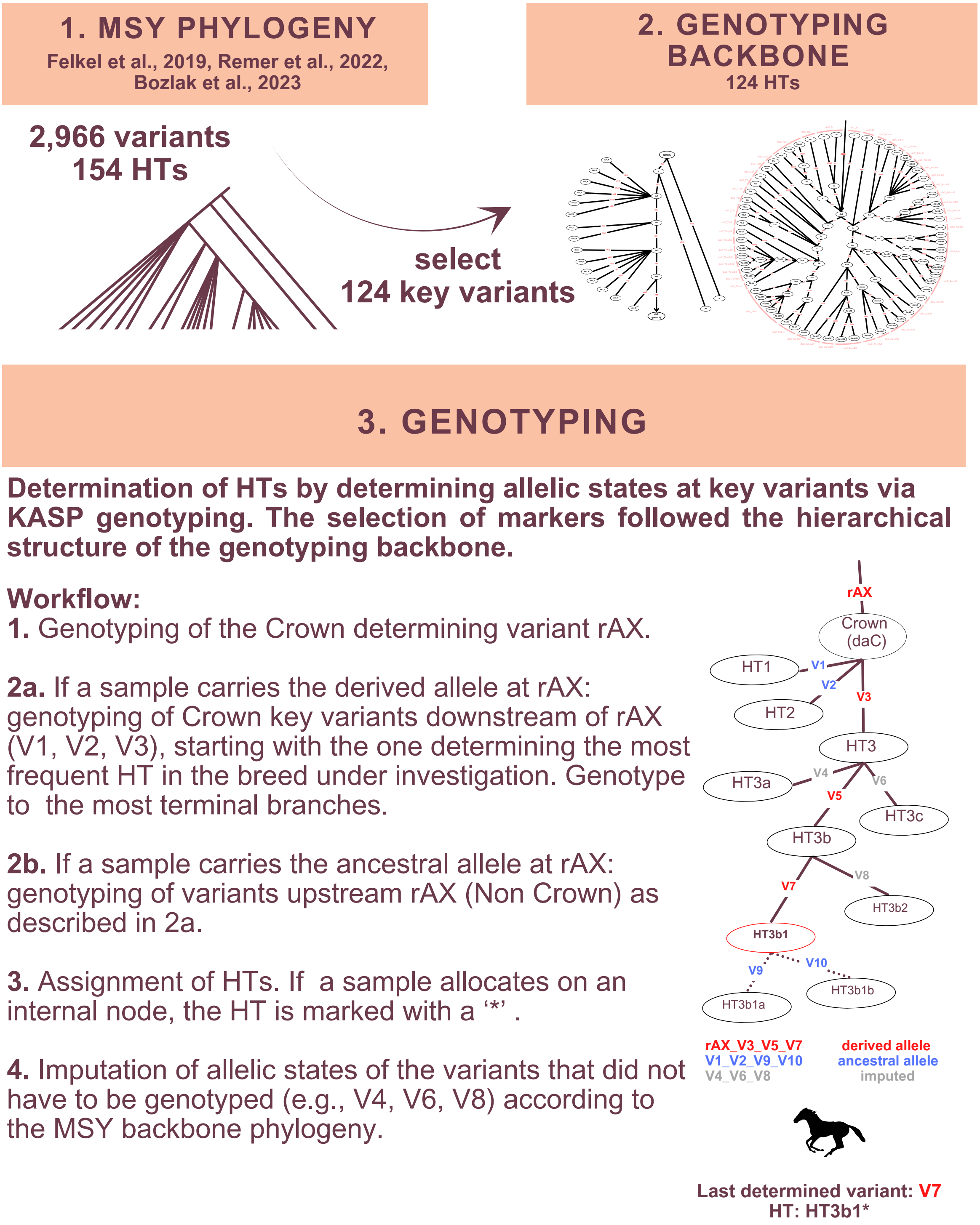

S3 Fig. Illustration of the genotyping workflow. For haplotype determination we adapted variants from the most recent horse Y phylogeny (1, 3, 4) and created genotyping backbones based on 124 selected 'key' variants, whose allelic states were determined with KASP genotyping. Genotyping was carried out hierarchically by first testing for Crown determining variant (rAX), followed by investigation of downstream and upstream variants depending on the results. In the genotyping process known frequencies of HTs in the breed of interest were considered.

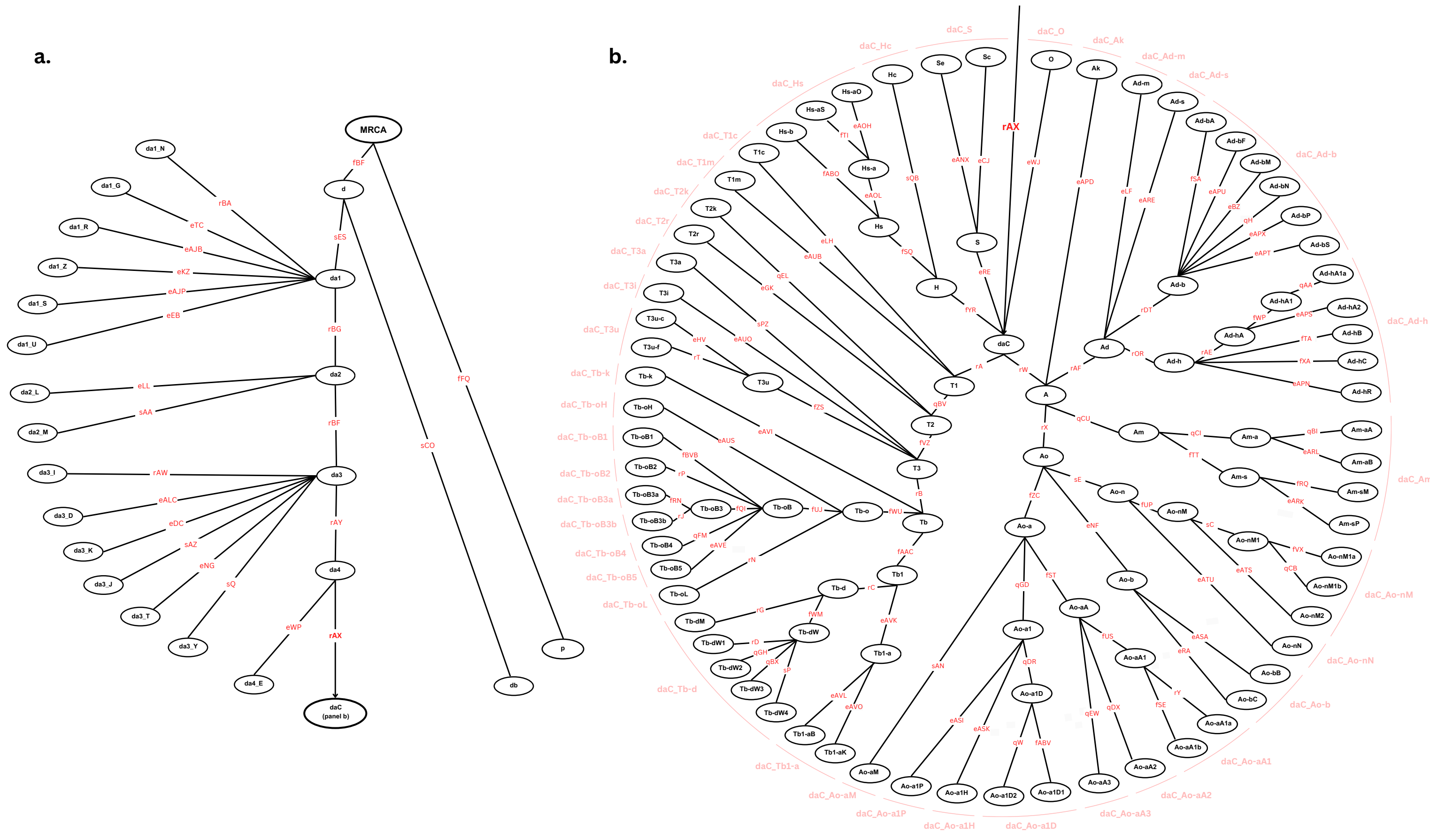

S4 Fig. MSY genotyping backbones. Downscaled MSY HT networks of Non Crown **(a)** and Crown **(b)** HG used for haplotype determination. HT topology is based on 124 variants ascertained by (1, 3, 4). Selected HT determining variants used for genotyping are denoted on each line in red and listed in Figure S2. Sub-haplogroups (sHGs) condensing HTs are colored with pink on the outer circle.

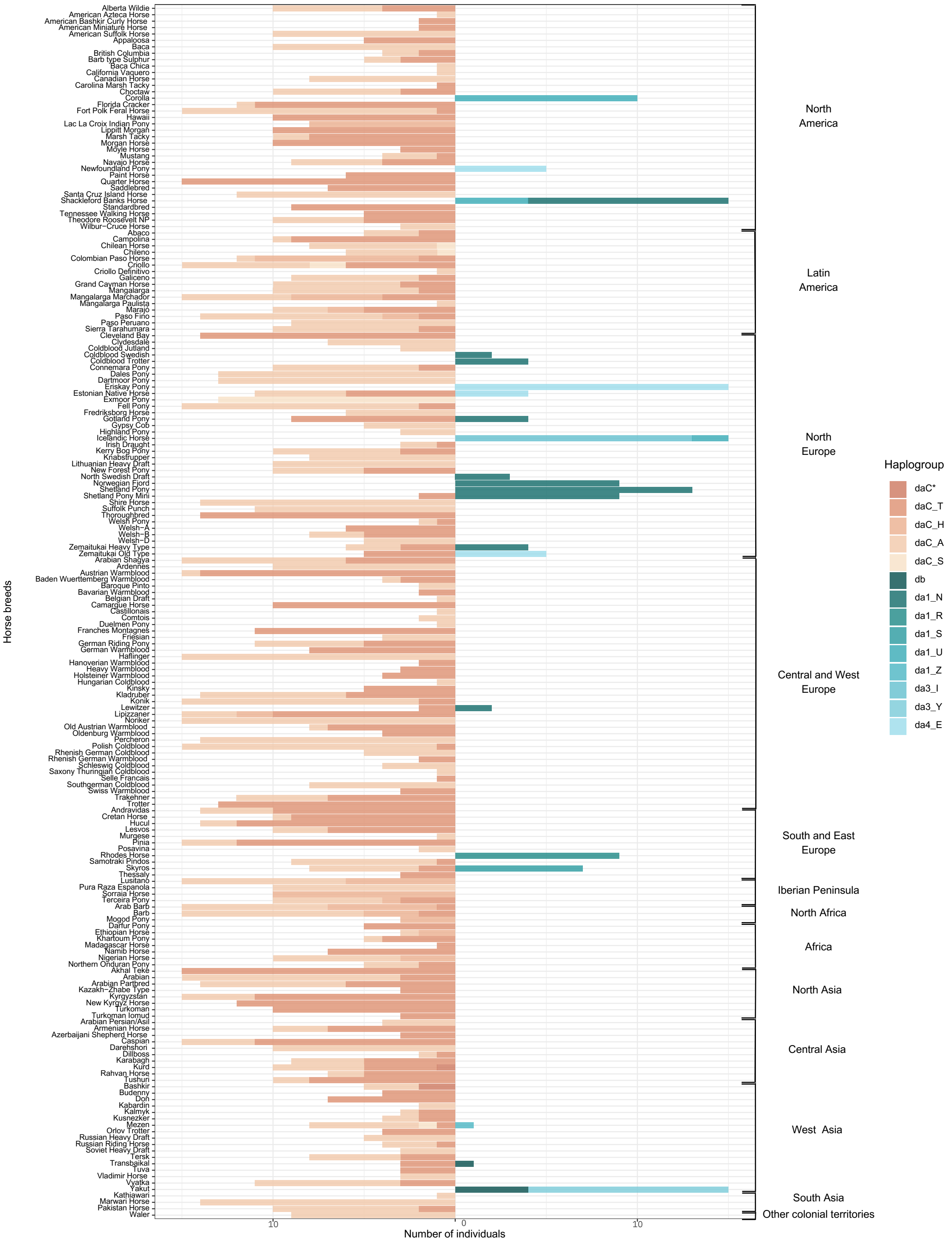

S5 Fig. Breed distribution. Horse breeds included in the dataset (189 breeds) are listed on the y axis, while horizontal bars on the x axis indicate number of individuals genotyped in each breed. Color code is according to Crown (pink shades) and outside the Crown (blue shades) haplogroups detected. Breeds are grouped according to their geographic origin, which is denoted on the right.

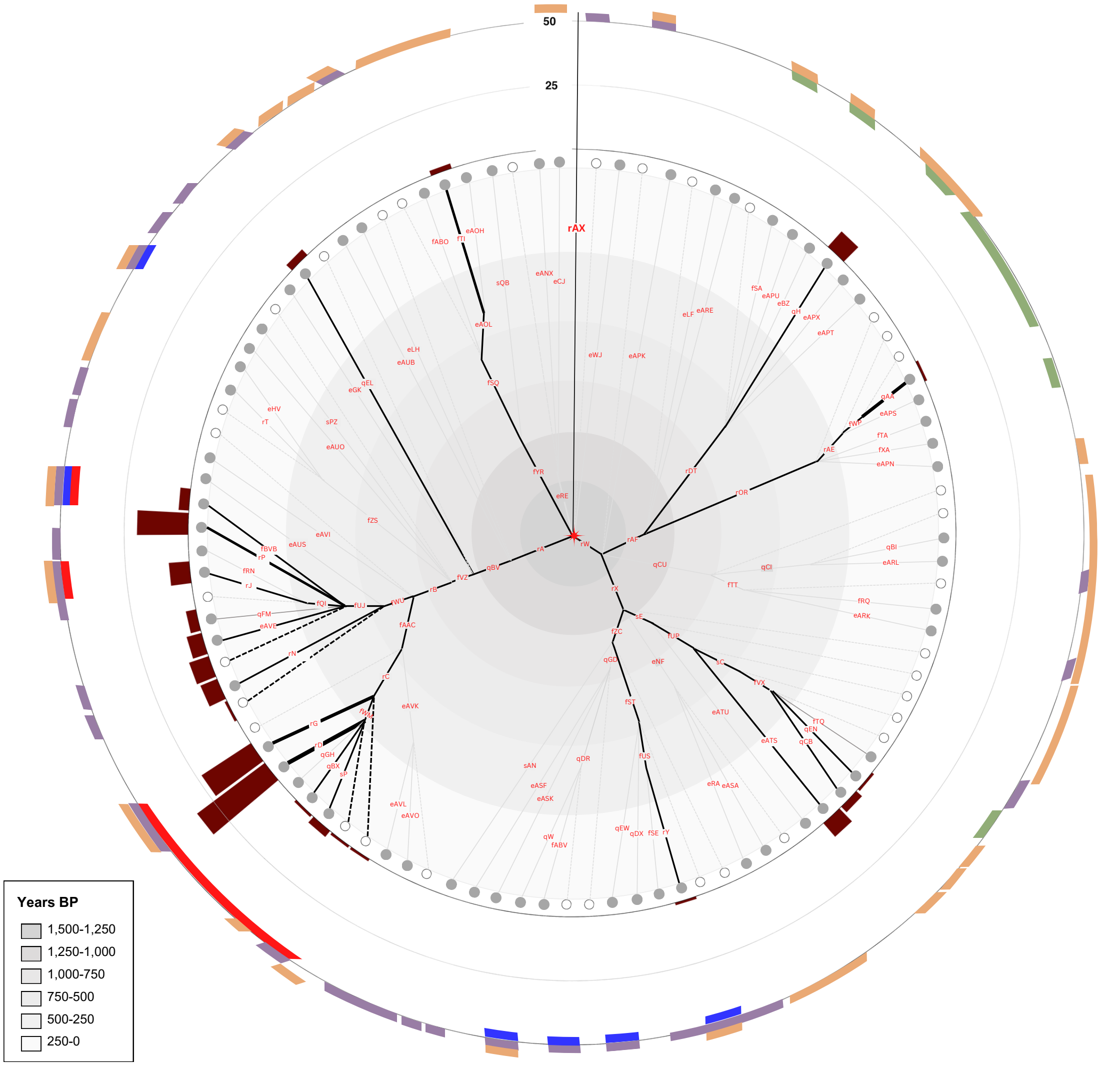

S6 Fig. MSY HTs detected in European and USA riding horses (n=146). Sunburst plots showing the frequency of HTs (sample set details in Dataset S1). The HT topology of the Crown (details in Fig. S4) is given in the center. '*HTs' allocated on internal nodes after genotyping are trailed with dashed lines towards a white dot from the corresponding internal node. Gray inner circles indicate time frames of branching points (Fig. 1b; Table S1, (5)). The bars on the inner circle denote detected HTs, with their frequency indicated as bar heights. Colored borders on the outer circle indicate HTs observed in Thoroughbreds (red), Arabians (blue), Coldbloods (green), Spanish influenced breeds (orange) and Local Asian breeds (purple) (details in Figure 2) while branches leading to the MRCA in the subset are bolded.

Supporting Table

S1 Table. Bayesian time estimates for the emergence of Crown haplogroups/sub-haplogroups. The time estimates were ascertained with BEAST (5), and are given as a mean of years before present (BP) for each node (basal branching point of haplogroup), with corresponding confidence intervals calculated from time interval ranges (given in years).

Node (haplogroup or subhaplogroup)	BEAST estimate (BP)	Confidence interval
daC	1495.09	± 221
daC_S	1073.53	± 300
daC_H	819.54	± 289
daC_Hs	513.42	± 213
daC_Hs-b	115.13	± 113
daC_Hs-a	380.70	± 180
daC_T1	1058.95	± 239
daC_T2	850.83	± 241
daC_T3	725.43	± 194
daC_T3a	172.92	± 130
daC_T3u	297.71	± 179
daC_T3u-f	164.96	± 117
daC_Tb	635.25	± 148
daC_Tb-o	487.43	± 132
daC_Tb-oB	377.19	± 115
daC_Tb-oB3	266.58	± 116
daC_Tb-oL	203.87	± 131
daC_Tb-1	448.15	± 144
daC_Tb-d	270.20	± 117
daC_Tb-dW	200.66	± 93
daC_Tb1-a	277.60	± 139
daC_Tb1-aK	203.12	± 122
daC_A	1253.15	± 202
daC_Ad	999.47	± 129
daC_Ad-b	435.88	± 184
daC_Ad_h	280.06	± 127
daC_Ad-hA	180.33	± 103
daC_Ad-hA1	130.78	± 83
daC_Am	677.74	± 230
daC_Am-s	552.31	± 221
daC_Am-a	333.52	± 200
daC_Ao	967.30	± 191
daC_Ao-n	783.98	± 216
daC_Ao-nM	633.28	± 200
daC_Ao-nM1	387.76	± 170
daC_Ao-nM1a	241.66	± 138
daC_Ao-b	510.32	± 285
daC_Ao-a	780.58	± 181
daC_Ao-a1	770,55	± 186
daC_Ao-a1D	368.90	± 193
daC_Ao-aA	428.08	± 150
daC_Ao-aA1	313.10	± 119

S2 Table. Ancestry Signatures. This table is related to Figure 4 and gives background information on ancestry signature determination. For each haplotype (HT) or sub-haplpogroup (sHG), a HT determining variant (DV) is given along with the rationale used for defining the ancestry signature. Number of individuals (Crown= 1,365) with corresponding HTs and breed group are given in the last column. Breed groups used for ancestry prediction are listed in black, as follows: Arabian (Ar), Thoroughbred (Tb), Coldblood (Cb), Spanish-influenced (Sp) and Local riding and light draft horses-Asia (L-As); breed groups that were not used for ancestry prediction are in grey: European and USA riding horses (Rd) and Local riding and light draft horses-Europe, USA and other colonial territories (L-EU). Details on sire lines are given in Dataset 1.

MSY HTs/sHG	DV	Ancestry signature	Ancestry signature defined in	Rationale for ancestry signature definition and remarks on observation of the HT	Number of individuals with given HT (and corresponding breed groups)
daC*	rAX	unexplored			3 (2 Cb, 1 L-As)
daC_A*	rW	unexplored			10 (1 Sp, 3 L-As; 6 L-EU)
daC_Ad*	rAF	unexplored			1 (L-EU)
daC_Ad-b*	rDT	Spanish/Coldblood	this article	detected in Coldblood breed and Spanish Colonial horses	67 (27 Cb, 21Sp; 19 L-EU)
daC_Ad-s	eARE	unexplored			4 (L-EU)
daC_Ad-bA	fSA	Spanish	this article	based on topology	20 (L-EU)
daC_Ad-bF	eAPU	Spanish	this article	based on topology and detected in Spanish Colonial horses	21 (12 Sp; 9 L-EU)
daC_Ad-bM	eBZ	Spanish/Coldblood	this article	based on topology, detected in Coldbloods	7 (5 Cb; 2 L-Eu)
daC_Ad-bN	qH	Spanish	this article	based on topology, noted in Kladruber, Friesian	10 (8 Rd, 2 L-EU)
daC_Ad-bP	eAPX	Spanish	this article	based on topology, described in Fell pony	4 (1 Sp; 3 L-EU)
daC_Ad-bS	eAPT	Coldblood	this article	detected in Coldblood breeds, characteristic for Shire horse	55 (28 Cb, 7 Sp; 20 L-EU)
daC_Ad-h*	rOR	Spanish/Coldblood	this article	based on topology, noted in numerous heavy draft horses and Spanish influenced breeds	59 (26 Cb, 12 Sp; 21 L-EU)
daC_Ad-hA*	rAE	Spanish/Coldblood	this article	based on topology, noted in heavy draft horses and Spanish influenced breeds	4 (1 Cb, 1 Sp; 2 L-EU)
daC_Ad-hA1*	fWP	Spanish/Coldblood	this article	based on topology, noted in heavy draft horses and Spanish influenced breeds	37 (27 Cb, 3 Sp; 7 L-EU)
daC_Ad-hA1a	qAA	Coldblood	this article	detected in Coldblood breeds, majority of Coldbloods carry this haplotype	44 (41 Cb,1 L-As; 1 Rd, 1 L-EU)
daC_Ad-hB	fTA	Coldblood	this article	detected in Coldblood breeds, characteristic for Noriker breed	5 (Cb)
daC_Ad-hC	fXA	Spanish	this article	based on topology, noted in Haflinger	10 (L-EU)
daC_Ad-hR	eAPN	Spanish	this article	based on topology, noted in Spanish Colonial horse	3 (Sp)
daC_Am*	rX	Spanish	this article	typical for Iberian, African and Latin American breeds	22 (20 Sp; 2 L-EU)
daC_Am-a*	qCI	Spanish	this article	typical for Iberian, African and Latin American breeds	5 (3 Sp; 2 L-EU)
daC_Am-aA	qBI	Spanish	Radovic et al., 2022; this article	first described in North African Barb horse, also noted in Iberian and Latin American breeds	5 (Sp)
daC_Am-aB	eARL	Spanish	Radovic et al., 2022; this article	first described in North African Barb horse, also noted in Iberian and Latin American breeds	2 (Sp)
daC_Am-s*	fTT	Spanish	this article	typical for Iberian, African and Latin American breeds	6 (5 Sp, 1 L-As)
daC_Am-sM	fRQ	Spanish	this article	typical for Iberian, African and Latin American breeds, first described in Mangalarga Marchador	15 (Sp)
daC_Am-sP	eARK	Spanish	this article	typical for Iberian, African and Latin American breeds, first described in Peruvian Paso horse	8 (Sp)
daC_Ao*	rX	Spanish	this article	found in Latin American breeds	3 (1 Sp, 2 L-As)

daC_Ao-n*	sE	Spanish	this article	noted in Abaco	1 (Sp)
daC_Ao-nM*	fUP	Spanish	this article	noted in Northern Onduran (Sudan) Pony, Ethiopian horse, Mangalarga	7 (1 Sp; 6 L-EU)
daC_Ao-nM1*	sC	Spanish	this article	based on topology; noted in Lusitano	2 (Sp)
daC_Ao-nM1a*	fVX	Spanish	this article	based on topology; noted in Kabardin horse	1 (L-As)
daC_Ao-nM1a1	fTQ	Coldblood	this article	detected in Coldblood breeds, typical for Noriker breed, carried by Haflingers	16 (13 Cb; 3 L-EU)
daC_Ao-nM1a2	qEN	Spanish	this article	based on topology; typical for Iberian horse breeds	20 (19 Sp; 1 Rd)
daC_Ao-nM1b	qCB	Spanish	this article	based on topology; noted in Kladruber horse	3 (Rd)
daC_Ao-nM2	eATS	Spanish	this article	based on topology; in Fredriksborg horse and Philippines horse	10 (3 Sp; 7 Rd)
daC_Ao-nN*	eATU	Spanish	this article	based on topology; noted in African Nigerian horse	3 (L-EU)
daC_Ao-b*	eNF	Spanish	this article	typical for Latin American breeds	2 (Sp)
daC_Ao-bC	eRA	Spanish	this article	typical for Latin American breeds	7 (Sp)
daC_Ao-bB	eASA	Spanish	this article	typical for Latin American breeds	12 (Sp)
daC_Ao-a*	fZC	West Asian	this article	noted in Marwari, Pakistan horse, Kabardin	11 (L-As)
daC_Ao-aA*	fST	West Asian	this article	noted in Caspian, Dareshori, Kyrgyzstan horses	4 (L-As)
daC_Ao-aA1a	rY	Arabian	Remer et al., 2022	most frequent Arabian haplotype, present in many Arabian sire lines	95 (41 Ar, 26 Sp, 19 L-As; 1 Rd, 8 L-EU)
daC_Ao-aA1b	fSE	Arabian	Remer et al., 2022	present in Siglavy db 1810. patriline	3 (Ar)
daC_Ao-aA2	qDX	West Asian	this article	based on topology; reported in Haflinger	2 (L-EU)
daC_Ao-aA3	qEW	Arabian/West Asian	Remer et al., 2022; this article	noted in Shagya Arabian, Caspian,Kyrgyzstan horses	3 (1 Ar, 2 L-As)
daC_Ao-a1D*	qDR	Arabian/West Asian	Remer et al., 2022; this article	some Arabian sire linjes, as well as SouthEast European local breeds	4 (1 Ar, 1 L-As; 2 L-EU)
daC_Ao-a1D1	fABV	West Asian	this article	based on topology; described in Duelmen Ponies and Konik horses	5 (L-EU)
daC_Ao-a1D2	qW	Arabian	Remer et al., 2022	present in many Arabian sire lines, reported in Arabian lines from Egypt,Poland, Quatar,…	30 (9 Ar, 7 Sp, 9 L-As; 5 L-EU)
daC_Ao-a1H	eASX	West Asian	this article	based on topology; noted in Hucul horse	1 (L-EU)
daC_Ao-a1P	eASI	West Asian	this article	based on topology; characterized in horses from Pakistan	5 (L-As)
daC_Ao-aM	sAN	West Asian	this article	based on topology; characterized in horses from India	10 (L-As)
daC_Tb1*	fAAC	West Asian	this article	based on topology; noted only in Gotland ponies	5 (L-EU)
daC_Tb1-a*	eAVK	West Asian	this article	noted in Asian and some SouthEast European breeds	36 (5 Cb, 23 L-As; 8 L-EU)
daC_Tb1-aK	eAVO	West Asian	this article	mainly noted in Asian and some SouthEast European breeds	1 (L-As)
daC_Tb1-aB	eAVL	West Asian	this article	noted in Asian and some SouthEast European breeds	4 (3 L-As; 1 L-EU)
daC_Tb-d*	rC	West Asian	Felkel et al., 2019; this article	based on topology; HT carried by Darley Arabian, 1700 and others; noted in North American breeds	8 (7 Sp; 1 Rd)
daC_Tb-dW*	fWM	Thoroughbred	Felkel et al., 2019	HT after Darley Arabian	4 (2 Cb, 1 L-As; 1 Rd)
daC_Tb-dW1	rD	Thoroughbred	Felkel et al., 2019	HT after the thoroughbred stallion Whalebone (3)	149 (44 Tb, 1 Cb, 15 Sp, 27 L-As; 33 Rd, 29 L-EU)
daC_Tb-dW2	qGH	Thoroughbred	Felkel et al., 2019; Remer et al., 2022	HT after Darley Arabian	2 (1 Rd, 1 L-EU)
daC_Tb-dW3	qBX	Thoroughbred	Felkel et al., 2019; Remer et al., 2022	the HT carried after Darley Arabian	1 (Rd)

daC_Tb-dW4	sP	Thoroughbred	Felkel et al., 2019	the HT carried after Darley Arabian	10 (4 Sp; 3 Rd, 3 L-EU)
daC_Tb-dM	rG	Thoroughbred	Felkel et al., 2019	HT after Darley Arabian, typical for Standardbred	46 (6 Tb, 4 Cb, 3 Sp, 4 L-As; 23 Rd, 6 L-EU)
daC_Tb-o*	fWU	unexplored			2 (1 Rd, 1 L-EU)
daC_Tb-oL	rN	West Asian	this article	based on topology; noted in Lipizzan horses	8 (7 Rd, 1 L-EU)
daC_Tb-oB*	fUJ	West Asian	this article	based on topology; noted in numerous local breeds	38 (8 L-As; 8 Rd, 22 L-EU)
daC_Tb-oB1	fBVB	Arabian/Thoroughbred/West Asian	Remer et al., 2022; Felkel et al., 2019; this article	widely distributed, HT carried by Byerley Turk (1680), noted in Arabians, Turkomans, local breeds from Western Asia	65 (2 Ar, 25 Tb, 9 Sp, 11 L-As; 4 Rd, 14 L-EU)
daC_Tb-oB2	rP	unexplored			21 (1 Sp; 20 Rd)
daC_Tb-oB3*	fQI	West Asian	this article	based on topology; noted in numerous, local breeds	2 (L-As)
daC_Tb-oB3a	fWY	West Asian	this article	noted in Akhal Teke and Turkoman horses	24 (L-As)
daC_Tb-oB3b	rJ	Thoroughbred	Felkel et al., 2019	HT carried by Godolphin Arabian (1724)	45 (17 Tb, 4 Cb, 7 Sp, 2 L-As; 8 Rd, 7 L-EU)
daC_Tb-oB4	qFM	unexplored			6 (4 Rd, 2 L-EU)
daC_Tb-oB5	eAVE	unexplored			6 (Rd)
daC_Tb-oH	eAUS	West Asian	this article	based on topology; noted in Hucul horse	7 (1 Cb; 6 L-EU)
daC_Tb-k	eAVI	unexplored			1 (L-As)
daC_Tb*	rB	West Asian	this article	noted in Tushuri, Akhal Teke, Pakistan horse, Turkoman	7 (L-As)
daC_T3u*	fZS	Spanish	this article	noted in Spanish Colonial breeds	4 (1 Cb, 2 Sp; 1 L-EU)
daC_T3u-f	rT	Spanish	this article	noted in local and Spanish Colonial breeds	25 (4 Sp; 21 L-EU)
daC_T3a	sPZ	Arabian	Remer et al., 2022	present in many Shagya Arabian sire lines and the line after the stallion Bairactar	25 (12 Ar, 1 Cb, 1Sp, 4 L-As; 7 L-EU)
daC_T3*	fVZ	West Asian	this article	mainly in local riding and light draft breeds	7 (4 L-As; 3 L-EU)
daC_T2r	eGK	West Asian	this article	Kyrgyzstan horse	1 (L-As)
daC_T2k	qEL	unexplored			3 (Rd)
daC_T2*	qBV	Spanish/West Asian	this article	noted in Spanish Colonial and West Asian breeds	13 (12 Sp, 1 L-As)
daC_T1m	eAUB	Spanish	this article	noted in Latin American breeds	10 (Sp)
daC_T1c	eLH	Spanish	this article	Colombian horse	1 (Sp)
daC_T1*	rA	Spanish/West Asian	this article	Kyrgyzstan horse and Spanish Colonial horse	11 (10 Sp, 1 L-As)
daC_Hs*	fSQ	Spanish	this article	typical for Iberian, African and Latin American breeds, also noted in Baroque type breeds	2 (L-EU)
daC_Hs-a	eAOL	Spanish	this article	typical for Iberian, African and Latin American breeds, also noted in Baroque type breeds	
daC_Hs-aS	fTI	Spanish	this article	typical for Iberian, African and Latin American breeds, also noted in Baroque type breeds	18 (16 Sp; 2 Rd)
daC_Hs-aO	eAOH	Spanish	this article	typical for Iberian, African and Latin American breeds, also noted in Baroque type breeds	8 (Sp)
daC_Hs-b	fABO	Spanish	this article	typical for Iberian, African and Latin American breeds, also noted in Baroque type breeds	34 (31 Sp; 3 L-EU)
daC_S*	eRE	Spanish	this article	based on topology; local breeds	12 (1 Cb; 11 L-EU)
daC_Se	eANX	Spanish	this article	based on topology; found in Exmoor Pony	2 (L-EU)
daC_Sc	eCJ	Spanish	this article	noted in Colonial breeds	4 (Sp)

Legends for Datasets

S1 Dataset (separate file). Sample table. Gives detailed information on 1,517 samples included in the study. The table contains Sample identification (ID), breed, country/region of breed, geographic region (Geographic group, according to Figure S2), remarks on sampled horse if available, as well as main and detailed breed groups (columns A-G). For samples with available pedigree, ancestors in their male tale lineage are listed (columns H-O). Details on genotyping results including Crown/Non Crown allocation, haplotype, most terminal variant with derived allele and sHG are in columns P-S. Column T provides information whether the sample was used for signature determination (Figure 2) while column U gives details on MSY ancestry determination and visualization. Determined ancestry signatures are given in column V. If a sample was used previously, the most recent study that included the sample is given. Last, availability and type of biosample (Biosample source), as well as sample responsibility (Sample contact) are provided.

S2 Dataset (separate file). Genotyping table. Provides information on 124 key variants screened in the dataset. For each variant ID, coordinates on LipY764 reference (3) are given along with the Ref/Alt Allele, the derived allelic state, variant type and the flanking regions used for primer construction. Additional notes on KASP genotyping and allelic signals of female controls are also given. Furthermore, the position of the variants in the MSY tree is provided: information if it is a Crown or Non Crown variant and which HT is determined by the variant. From Column M onwards, the genotyping results of the 1,517 samples included in the study are given as Ref/Alt allelic states (0/1) with the Sample IDs corresponding to the S1 Dataset, denoted in the first row.

S3 Dataset (separate file). Dating table. The IDs of published NGS samples (4) used for construction of the parsimony tree (Figure 1) are given with their breeds, Crown/Non Crown allocation and haplotype. Previously determined Thoroughbred and Arabian specific HTs (1, 3) are indicated. Likewise, details on resequencing approach are listed in Source column: WGS (whole genome sequencing) or TES (target enriched sequencing).

SI References

1. V. Remer, *et al.*, Y-Chromosomal Insights into Breeding History and Sire Line Genealogies of Arabian Horses. *Genes (Basel).* **13** (2022).
2. Á. Maróti-Agóts, L. Zöldág, N. Solymosi, B. Egyed, Effect of different sampling methods on cattle mtDNA phylogenetic studies. *B. Abstr. 59th Annu. Meet. Eur. Assoc. Anim. Prod. Vilnius, Lith. August 24th-27th, 2008* **15**, 130 (2008).
3. S. Felkel, *et al.*, The horse Y chromosome as an informative marker for tracing sire lines. *Sci. Rep.* **9**, 1–12 (2019).
4. E. Bozlak, *et al.*, Refining the evolutionary tree of the horse Y chromosome. *Sci. Rep.* **13**, 1–13 (2023).
5. R. Bouckaert, *et al.*, BEAST 2.5: An advanced software platform for Bayesian evolutionary analysis. *PLoS Comput. Biol.* **15**, 1–28 (2019).
